# Identification of Potential Candidate Genes of Oral Cancer in Response to Chronic Infection With *Porphyromonas gingivalis* Using Bioinformatical Analyses

**DOI:** 10.3389/fonc.2019.00091

**Published:** 2019-02-21

**Authors:** Fengxue Geng, Qingxuan Wang, Chen Li, Junchao Liu, Dongmei Zhang, Shuwei Zhang, Yaping Pan

**Affiliations:** ^1^Department of Periodontics, School of Stomatology, China Medical University, Shenyang, China; ^2^State Key Laboratory of Oral Disease, School of Stomatology, Sichuan University, Chengdu, China

**Keywords:** *Porphyromonas gingivalis*, chronic infection, inflammation, oral cancer, bioinformatics

## Abstract

Recent investigations revealed the relationship between chronic periodontitis, *Porphyromonas gingivalis* and cancer. However, host genes that change in response to chronic infection with *P. gingivalis* and may contribute to oral cancer have remained largely unknown. In the present study, we aimed to comprehensively analyze microarray data obtained from the chronic infection model of immortalized oral epithelial cells that were persistently exposed to *P. gingivalis* for 15 weeks. Using protein-protein interaction (PPI) networks and Ingenuity Pathway Analysis (IPA), we identified hub genes, major biological processes, upstream regulators and genes potentially involved in tumor initiation and progression. We also validated gene expression and demonstrated genetic alteration of hub genes from clinical samples of head and neck cancer. Overall, we utilized bioinformatical methods to identify *IL6, STAT1, LYN, BDNF, C3, CD274, PDCD1LG2*, and *CXCL10* as potential candidate genes that might facilitate the prevention and treatment of oral squamous cell carcinoma (OSCC), the most common type of head and neck squamous cell carcinoma (HNSCC).

## Introduction

Periodontitis is a chronic infectious disease with high global prevalence (1). The persistent immune-inflammation response initiated by periodontal pathogens not only causes local damage in oral sites, but also promotes systemically chronic inflammation such as the pathogenesis of cancer (2). Based on the latest meta-analysis, periodontal disease is positively associated with the risk of pancreatic, lung, head and neck cancers (3). Furthermore, a cohort study with 10-year follow-up recently revealed a significant relationship between periodontitis and cancer mortality (4).

*Porphyromonas gingivalis*, one of the best characterized pathogens of periodontitis, is regarded as a keystone pathogen because of its ability to induce increased community biomass and manipulate subversion of host immune responses (2, 5). With a variety of virulence factors, *P. gingivalis* can interact with host cells for successful internalization and intracellular survival, which consequently results in chronic infection (6). Of note, *P. gingivalis* is now accepted as a risk factor of cancer such as oral squamous cell carcinoma (OSCC), the most common type of head and neck squamous cell carcinoma (HNSCC) (6–8). To date, several studies have shown that acute *P. gingivalis* infections (i.e., those lasting no more than 72 h) affected cell division, apoptosis, migration and invasion in gingival epithelial cells or OSCC cells (2). However, since cancer can be a complicated and gradual process (9), little is known about genes that contribute to OSCC in a microenvironment characterized by chronic *P. gingivalis* infection. To better mimic the infection mode *in vivo*, we established a novel model system using human immortalized oral epithelial cells (HIOECs) persistently exposed to *P. gingivalis* at a multiplicity of infection (MOI) of 1 (10). Furthermore, we reported the promoting effect of chronic infection by *P. gingivalis* on cellular morphology, cell proliferation, migration and invasion of HIOECs. Also, using microarray analysis and relevant validation of changes in expression, we identified genes that may be involved in the tumorigenic-like transformation of HIOECs induced by *P. gingivalis* (10). For further investigation, in this study, we aimed to systematically analyze the differentially expressed genes (DEGs) from previous microarray data. To our knowledge, this work is the first to show and analyze DEGs in response to *P. gingivalis* infections lasting up to 15 weeks. We identified *IL6, STAT1, LYN, BDNF, C3, CD274, PDCD1LG2*, and *CXCL10* as candidate genes associated with the initiation and progression of oral cancer induced by *P. gingivalis* infection.

## Methods

### Establishment of Cellular Model and Microarray Analysis

The establishment of a novel cellular model of HIOECs persistently exposed to *P. gingivalis* was described previously (10). In short, *P. gingivalis* ATCC 33277 was routinely recovered on brain heart infusion (Difco Laboratories, MI, USA) agar plates and cultured in brain heart infusion broth medium supplemented with 0.5% yeast, 0.1% menadione, and 1% hemin at 37°C in an anaerobic environment (80% N_2_, 10% H_2_, and 10% CO_2_). HIOECs were kindly provided by Dr. Wantao Chen from Key Laboratory of Shanghai Oral Medicine, Ninth People's Hospital, Shanghai Jiao Tong University. HIOECs were cultured in Defined Keratinocyte-SFM (GibcoTM, Thermo Fisher Scientific Inc., MA, USA) with growth supplement in a humidified atmosphere (37°C, 5% CO_2_). To establish the infection model, actively growing HIOECs in six-well plates were infected with *P. gingivalis* at an MOI of 1 for 24 h each time. The infection was repeated at each passage. HIOECs without *P. gingivalis* infection were cultured at the same time as a control.

When the HIOECs infected by *P. gingivalis* showed significantly increased tumorigenic properties after 15 weeks, the total RNA of non-infected HIOECs and infected HIOECs was extracted with an RNAiso Plus kit (TaKaRa, Dalian, China). As described before (10), the microarray (Affymetrix) that could simultaneously detect coding and non-coding genes was conducted at the OEbiotech Corporation (Shanghai, China). An Affymetrix Scanner 3000 (Affymetrix) and Affymetrix GeneChip Command Console (version 4.0, Affymetrix) software was applied for array scan and raw data exaction, respectively. Expression Console (version 1.3.1, Affymetrix) software was used for robust multi-array average (RMA) normalization. DEGs were identified using GeneSpring software (version 13.1; Agilent Technologies). Student's *t*-test was used for statistical analysis. The threshold set for aberrantly regulated genes was a fold change ≥2.0 and a *P* < 0.05. The *P*-value was adjusted by multiple testing using the Benjamini-Hochberg false discovery rate (FDR) procedure and a standard threshold of 5% was selected for declaring significance (11). In the present study, we focused on the coding genes that were aberrantly expressed. The microarray dataset has been uploaded to NCBI GEO platform (GSE87539).

### Data Analyses

To construct the protein-protein interaction (PPI) network, the 423 differentially expressed coding genes from the present dataset were imported into the STRING (version 10.5) online database (https://string-db.org) and filtered with the interaction score of high confidence (0.700) (12). The network was then visualized with Cytoscape software (version 3.6.0). Using cytoHubba, a plugin of Cytoscape, 10 hub genes were calculated and ranked by “Radiality.” Gene Ontology analysis was performed with BiNGO from Cytoscape software. The Ingenuity Pathway Analysis (IPA) was performed by Genery Bio Corporation (Shanghai, China). IPA platform provided regulator networks of biological functions and diseases based on a large-scale causal network derived from the Ingenuity Knowledge Base (13). In this study, the upstream regulators and the enriched gene functions of the present cellular model were analyzed by IPA. Fisher's Exact Test was used for statistical analysis.

**Figure 1:**
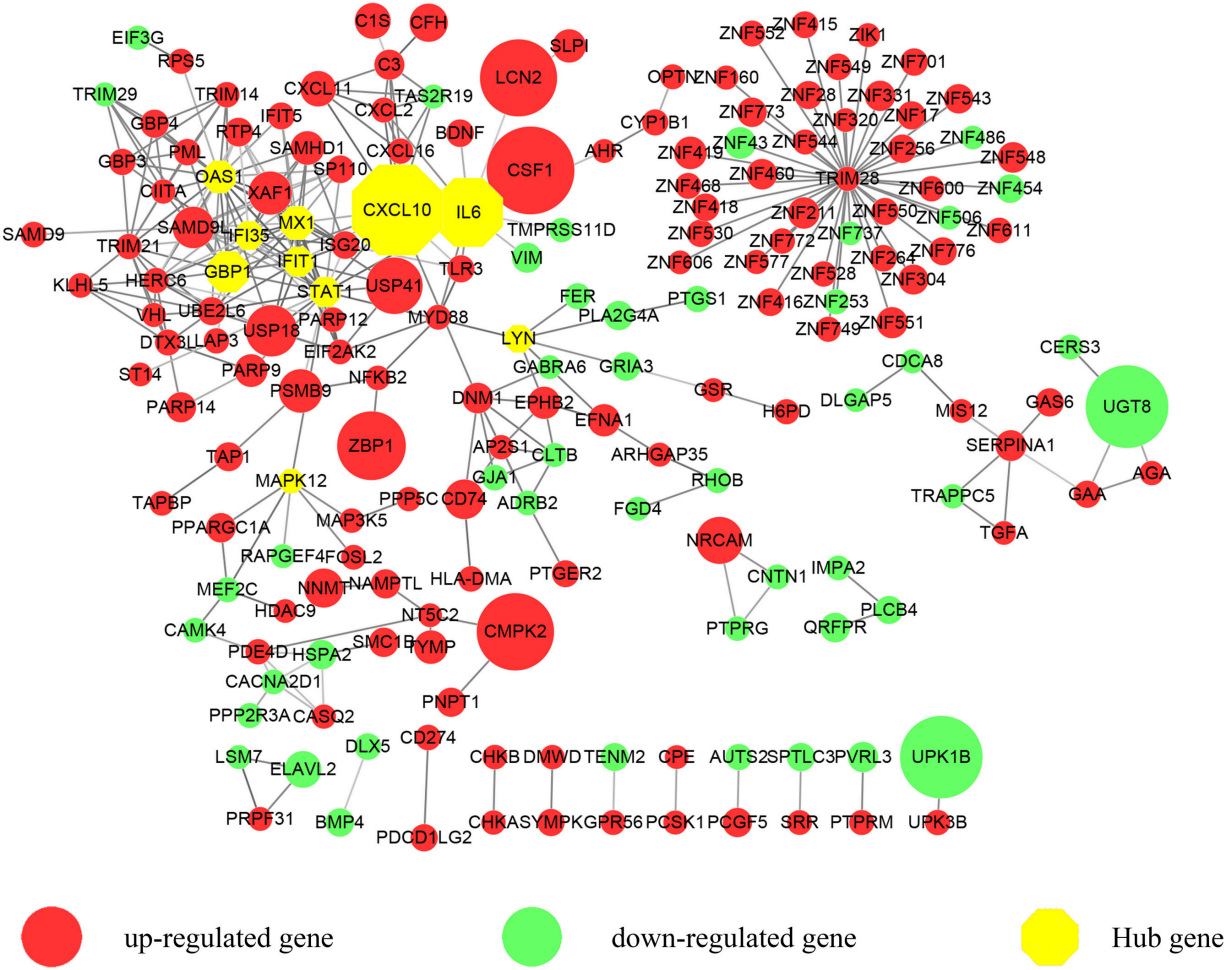
A protein-protein interaction (PPI) network exported from STRING was visualized with Cytoscape. 183 nodes and 309 edges were displayed. Up-regulated genes are shown in red while down-regulated genes are shown in green. Larger node sizes correspond to higher fold changes of DEGs. Edges were shown in gray from light to dark according to the combined score (from low to high). The hub genes were emphasized with octagon shapes in yellow.

To validate the expression of DEGs in clinical samples, we firstly screened the expression of the 10 hub genes by applying the Oncomine^TM^ platform (https://www.oncomine.org/resource/login.html). Oncomine^TM^ was a cancer microarray database which facilitated to explore genome-wide expression analyses in various types of cancer as well as respective normal tissues. Specifically, a variety of cancer subtypes were also available (14). We selected seven datasets of OSCC, one of the important types of HNSCC. The search term of each gene was consistent with the gene symbol shown in the Figure 1. A *P*-value < 1E-4 and a fold change > 2 were considered as the threshold with gene ranking in the top 10%. Further, we selected one of the datasets (Peng Head-Neck, 79 samples, 18148 measured genes) to find the specific fold change of hub genes. The *P*-value was provided by the program (Oncomine^TM^). cBioPortal database was used to integrate multidimensional cancer genomic data, such as somatic mutations, DNA copy-number alterations (CNAs) and mRNA (15). The hub genes validated by Oncomine^TM^ were further analyzed for genetic alterations by using cBioPortal (http://www.cbioportal.org/). A network containing the hub genes and the most frequently altered neighbor genes was also produced by cBioPortal. Biological interactions were derived from public pathway databases including PANTHER, Reactome, pid, Phosphosite, HumanCyc, CancerRxGene and KEGG Drug.

## Results

### PPI Network Construction and Identification of Hub Genes

We focused on the coding genes in the present study. Among the 423 DEGs (fold change ≥ 2, *P* < 0.05), 296 genes were up-regulated while 127 genes were down-regulated. As shown in Figure 1, we first developed a PPI network. Three hundred and sixty-five of molecules were identified with STRING (PPI enrichment *P* < 1.0e−16). Disconnected nodes were hidden. As emphasized with a yellow octagon sign, *STAT1, CXCL10, MX1, IFIT1, GBP1, IL6, OAS1, MAPK12, LYN*, and *IFI35* were identified as the hub genes of the network. *STAT1, MX1, IFIT1, GBP1, OAS1*, and *IFI35* were associated with the most significant module. Hub gene ranks are shown in Supplementary Figure 1.

### Gene Ontology Analysis

**Figure 2:**
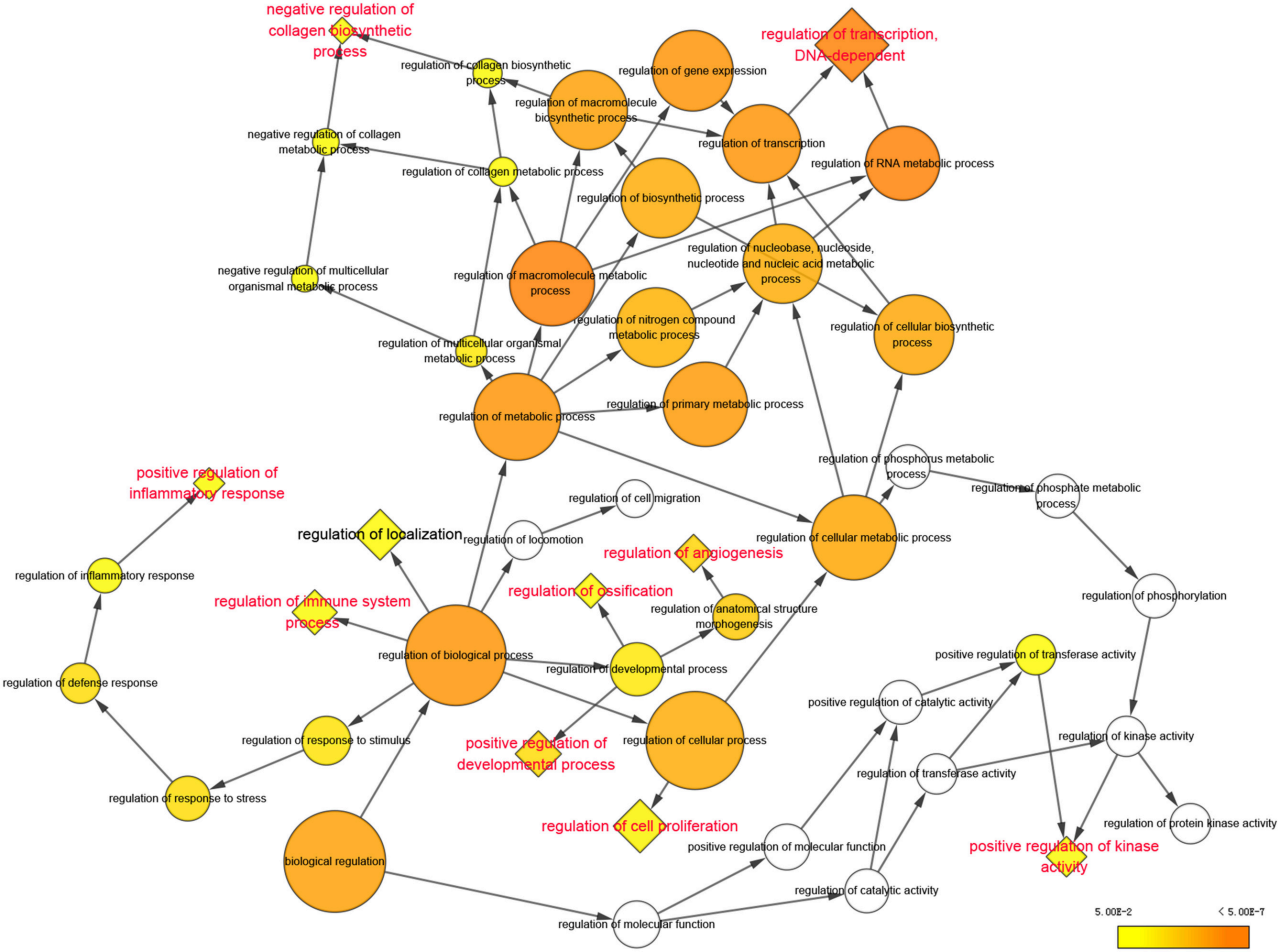
Significantly enriched biological processes of Gene Ontology were displayed in colored nodes. The nodes color got increasingly more orange for more significant *P*-values. The uncolored nodes that were not overrepresented were considered as the parents of the overrepresented categories further down. The size of the node depended on the number of genes annotated in the node.

According to the analysis with Cytoscape software, statistically overrepresented biological processes are shown in Figure 2 as colored nodes, which range from yellow to orange based on *P*-value. Nodes such as “regulation of metabolic process,” “regulation of biological process” and “regulation of cellular process,” though presented in larger size and darker orange, were merely the results of the constitutive genes at nodes further down each branch (16); we chose to focus our analyses on these nodes. Nine enriched biological processes, highlighted in red in Figure 2, include: “regulation of transcription, DNA-dependent,” “negative regulation of collagen biosynthetic process,” “positive regulation of inflammation response,” “regulation of immune system process,” “positive regulation of development process,” “regulation of ossification,” “regulation of angiogenesis,” “regulation of cell proliferation” and “positive regulation of kinase activity.”

### Upstream Regulators

**Figure 3:**
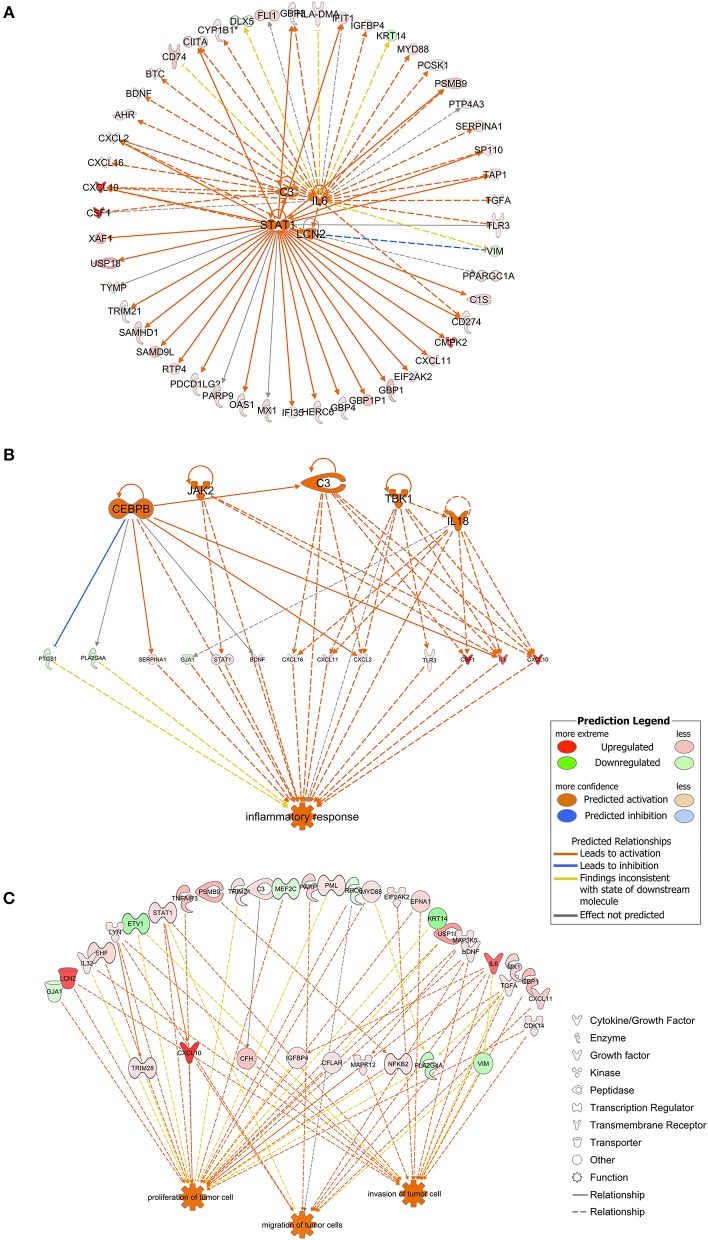
**(A)** The four molecules shown in the middle were considered as the upstream regulators and to regulate the surrounding genes. As shown in orange, *STAT1, C3, IL6*, and *LCN2* were predicted to be activated in the present model. Full lines indicated the direct relationships while dashed lines indicated indirect relationships. **(B)** Inflammation-associated genes were highlighted. Genes containing *CEBPB, JAK2, C3, TBK1*, and *IL18* were predicted to be activated and to regulate genes downstream. Full lines indicated direct relationships while dashed lines indicated indirect relationships. **(C)** Genes possibly involved in tumor cell proliferation, migration and invasion. Full lines indicated direct relationships while dashed lines indicated indirect relationships.

As shown in Figure 3A, *STAT1, C3, IL6*, and *LCN2* were predicted to function as upstream regulators in the present model. *STAT1* was predicted to directly up-regulate genes such as *CXCL10, IFIT1, GBP1, OAS1, IFI35, CD274*, and *PDCD1LG2*. *IL6* was predicted to up-regulate downstream genes such as *CD274, TGFA, SERPINA1, MYD88*, and *BDNF*. *C3* was predicted to up-regulate genes such as *CSF1, CXCL16*, and *CXCL2*. *LCN2* was predicted to up-regulate *CXCL10* and *CXCL2* and to down-regulate *VIM*. Among the downstream regulators, *CXCL10* was up-regulated by all four upstream regulators.

### Analysis of Biological Functions

Genes associated with inflammatory response were analyzed using the IPA platform and are shown in Figure 3B. *SERPINA1, STAT1, BDNF, CXCL16, CXCL11, CXCL2, TLR3, CSF1, IL6*, and *CXCL10* were up-regulated while *PTGS1, PLA2G4A*, and *GJA1* were down-regulated based on the present dataset. *CEBPB, JAK2, TBK1*, and *IL18*, which did not belong to the dataset, were predicted to be activated according to the IPA platform. As shown in Figure 3B, *CEBPB* directly activated the expression of *SERPINA1, CXCL2, IL6*, and *C3* and inhibited the expression of *PTGS1*.

Based on the results of the IPA platform, we discovered a series of genes that may be involved in regulation of tumorigenic properties of *P. gingivalis*-infected HIOECs. Most of the genes listed in Figure 3C were associated with cell proliferation and/or invasion, while genes associated with cell migration were relatively fewer in number. Genes such as *LCN2, LYN, STAT1, BDNF, IL6, CXCL10*, and *CDK14* were related to cell proliferation and cell metastasis.

### Validation of Hub Genes in Clinical Samples

**Figure 4:**
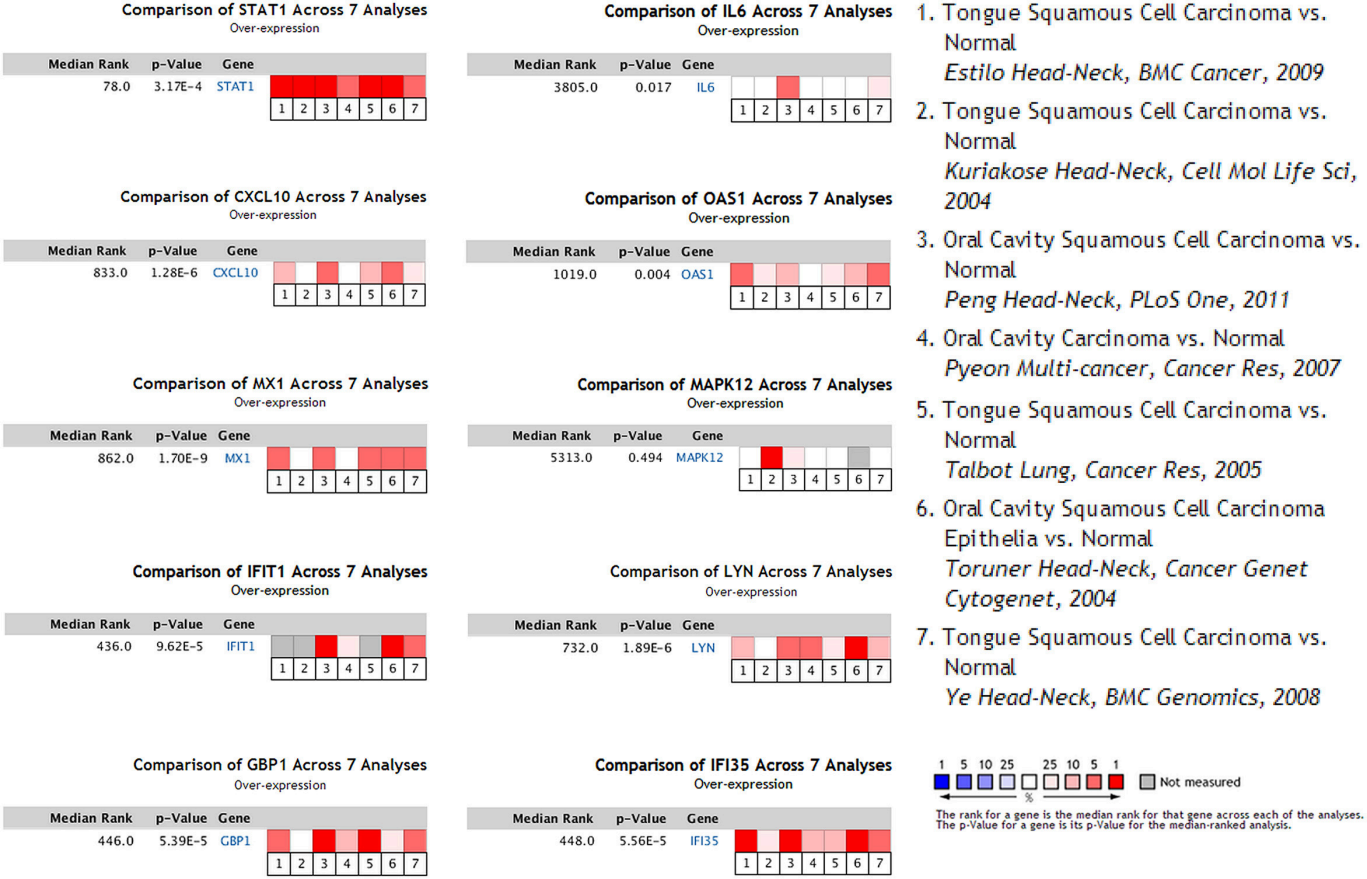
Seven OSCC datasets were selected and the expression of the hub genes was compared across the seven datasets. Among the datasets, three of them were obtained from general OSCC samples while the other four belonged to tongue squamous cell carcinoma, the most common subtype of OSCC. Normal tissues were selected as the control. Values above the average were considered over-expressed hub genes (red).

**Figure 5:**
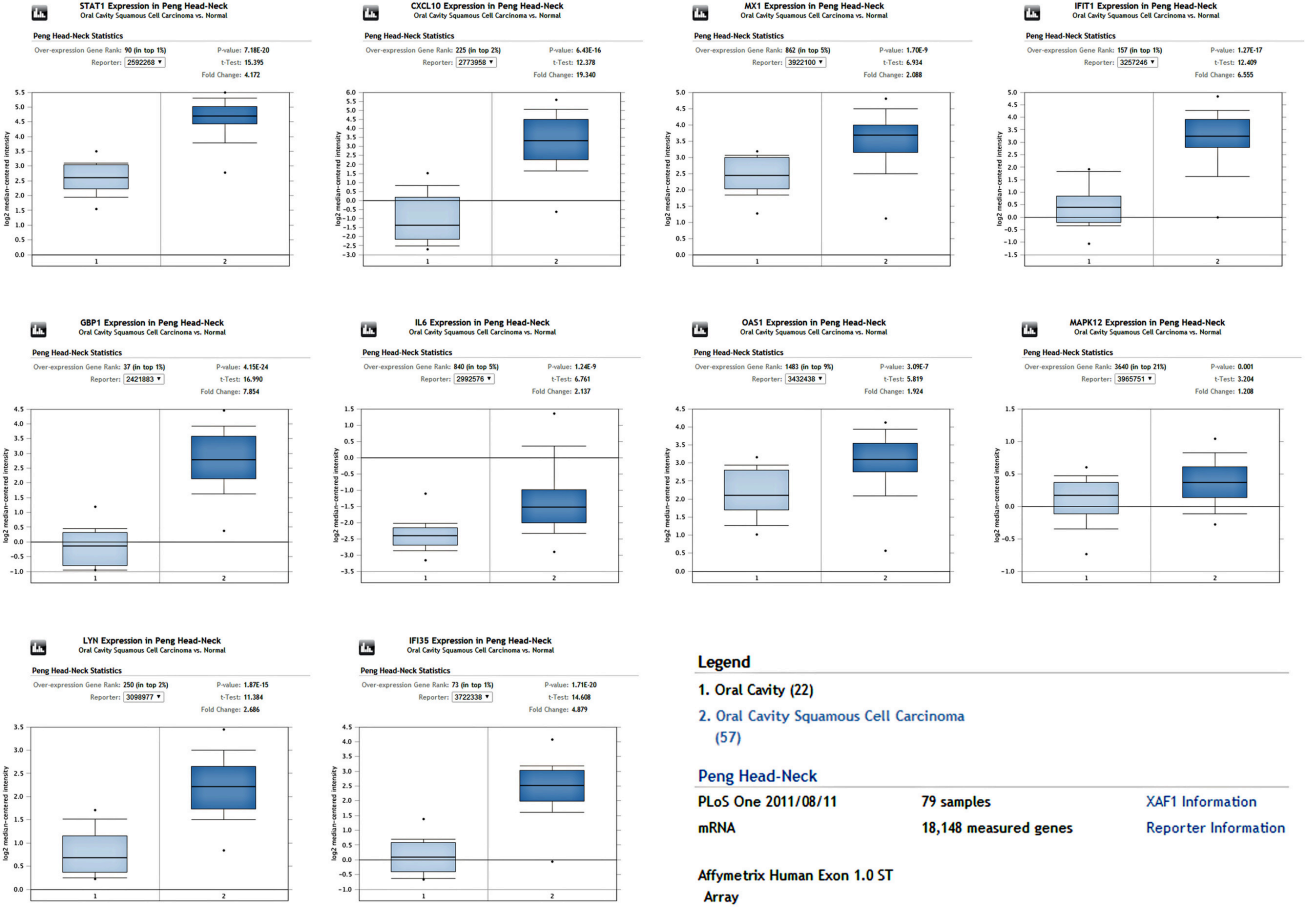
The expression of hub genes in one OSCC dataset containing 79 samples. All hub genes were overexpressed in the OSCC tissues compared to the control (*P* < 0.05).

To determine whether the DEGs identified in response to chronic infection by *P. gingivalis* were also aberrantly expressed in oral cancer, we chose to validate the expression of hub genes in clinical samples of OSCC from Oncomine^TM^. With the exception of *MAPK12*, most genes were found to be generally over-expressed in OSCC samples compared with the normal samples (*P* < 0.05) (Figure 4). Notably, *STAT1, GBP1, OAS1, IFI35*, and *LYN* were overexpressed in more than 6 datasets. In one specific OSCC dataset, all hub genes were significantly up-regulated in oral cavity squamous cell carcinoma samples compared to control samples (*P* < 0.05) (Figure 5).

### Genetic Alterations of Validated Genes and the Relative Network

**Figure 6:**
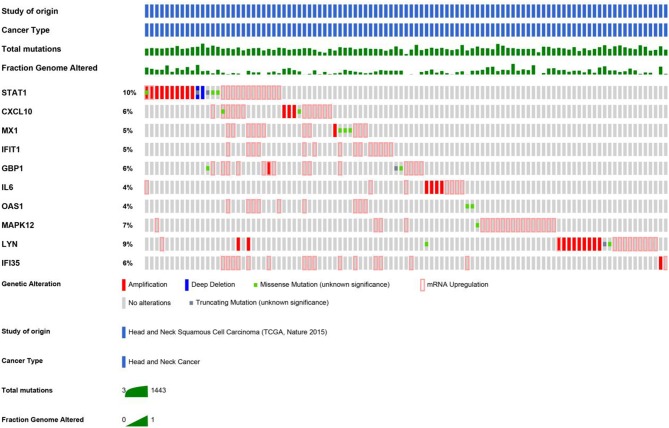
As shown, a study from HNSCC (TCGA, Nature 2015) was selected to analyze the genetic alterations of hub genes. The total mutations and fraction of the genome that was altered were also displayed, respectively.

**Figure 7:**
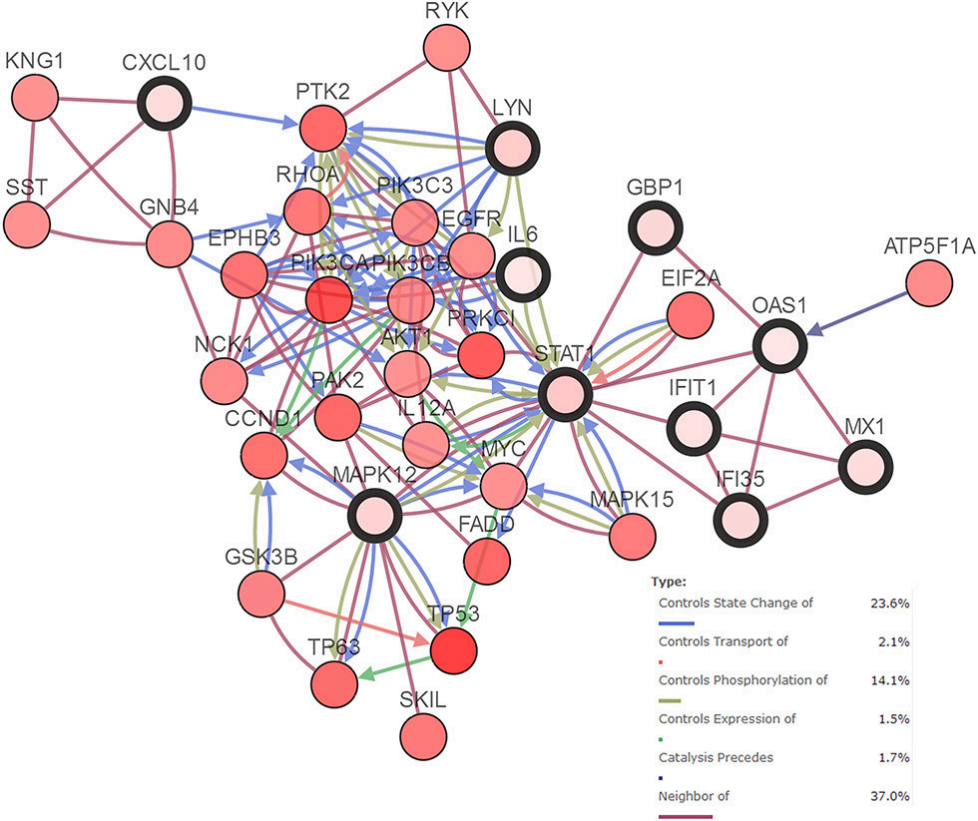
Network of hub genes (shown with thick border) and altered neighbor genes. Relationship among the genes included “Controls State Change of,” “Controls Transport of,” “Controls Phosphorylation of,” “Controls Expression of,” “Controls precedes,”and “Neighbor of”.

An HNSCC dataset provided by The Cancer Genome Atlas (TCGA) with 279 samples was used to analyze genetic alterations. As shown in Figure 6, the 10 hub genes were generally altered in 37% of the queried samples. Specifically, the gene set altered from 4 to 10% contained amplification, deep deletion, missense, mRNA up-regulation and truncation mutations. Using cBioPortal, we constructed a network that contained the hub genes and other frequently altered neighbor genes (Figure 7).

### Potential Candidate Genes

**Figure 8:**
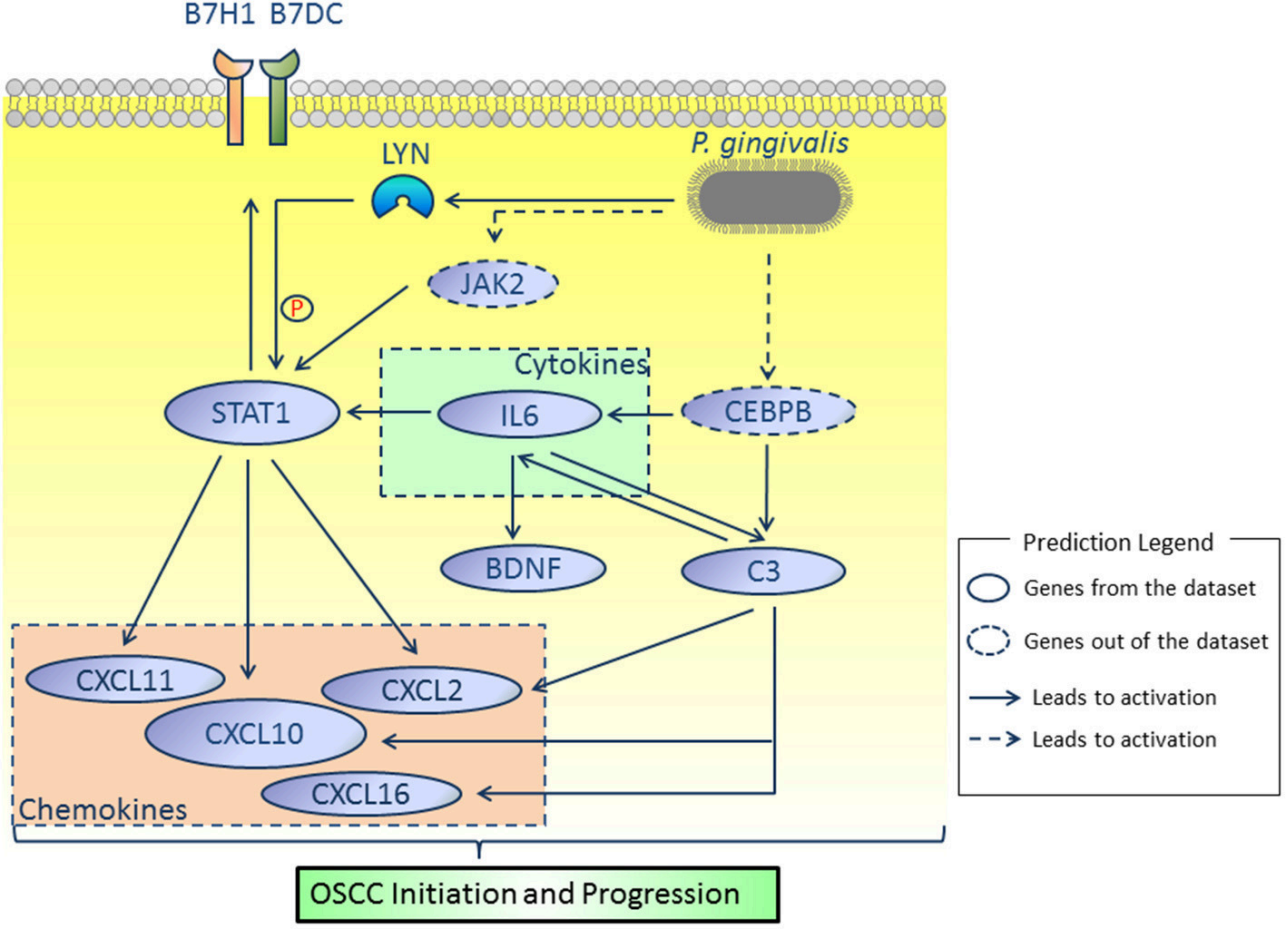
Proposed model. According to our results, *IL6, STAT1, LYN, BDNF, C3, CD274, PDCD1LG2*, and *CXCL10* were considered as key molecules involved in OSCC initiation and progression induced by *P. gingivalis*. *JAK2* and *CEBPB*, which were not identified in the present dataset, were predicted by the IPA platform and are shown in dotted lines. All relationships were based on IPA and cBioPortal database.

By comprehensively analyzing the data shown in Figures 1, 3, 4, 7, genes including *IL6, STAT1, LYN, BDNF, C3, CD274, PDCD1LG2*, and *CXCL10* were considered to be potential candidate genes and are presented in the proposed mode pattern in Figure 8. To be consistent with a previous study (2), *CD274* and *PDCD1LG2* are shown as *B7H1* and *B7DC*, respectively, in Figure 8. In the proposed diagram, *IL6, STAT1, LYN*, and *CXCL10* were hub genes while *C3, IL6*, and *STAT1* were activated as the upstream regulators of the present dataset. *CEBPB* and *JAK2*, which were predicted as upstream regulators, are also shown in the network. Additionally, chemokines including *CXCL2, CXCL11, CXCL16* were involved as possible downstream molecules. All candidate genes were associated with inflammation and/or tumors. Additional reasons for selecting these genes and their molecular functions in inflammation and tumors are discussed below.

## Discussion

It has been increasingly accepted that OSCC is the most relevant cancer type associated with oral bacterial infections (7). To date, several studies have reported the promoting effects of *P. gingivalis* on OSCC initiation and progression (10, 17–23). However, the mechanism of chronic infection by *P. gingivalis* in OSCC is not clearly understood. In the present study, we mined microarray data obtained from a cellular model of oral epithelial cells that were infected by *P. gingivalis* for 15 weeks. We first identified hub genes and found that DEGs were enriched in biological processes such as “positive regulation of inflammation response,” “regulation of immune system process,” “regulation of angiogenesis” and “regulation of cell proliferation.” Then, we specifically analyzed the upstream regulators and DEGs in tumor cell proliferation, migration, and invasion using the IPA platform. We previously validated some of the microarray data by qPCR (10). In the present study, using validation with datasets from clinical samples, we confirmed that some of the DEGs (hub genes) were also aberrantly expressed in OSCC clinical specimens.

Inflammation is well-accepted as a hallmark of tumorigenesis (24). Cytokines and chemokines play important roles in tumor initiation and progression. Cytokines such as *IL6* promote tumor initiation by elevating intracellular reactive oxygen species (ROS) and reactive nitrogen intermediates (RNI) as well as causing epigenetic alteration of certain genes. Furthermore, cytokines facilitate tumor progression by activating tumorigenic-related transcription factors (25). In turn, activated transcription factors induce the production of chemokines, which results in continuous tumor-associated inflammation (25). Among the genes listed in Figure 3, those such as *IL6* (cytokine) and *CXCL10* (chemokine) were shown to be involved in the inflammatory response (Figure 3B) and are positively related to cell proliferation and metastasis (Figure 3C). Interleukin (*IL6*), a tumor promoting cytokine in various types of cancer including OSCC (25–27), may function as a central molecule in the proposed network (Figure 8). Importantly, *IL6* was identified as one of the most promising predictors for early diagnosis and prognosis of tongue squamous cell carcinoma, the most common type of OSCC with high risk of local invasion and recurrence (27, 28).

We paid specific attention to the relationship between *CEBPB* and *IL6*. It is well-known that *IL6* is one of the major cytokines involved in *P. gingivalis* infection and periondontitis initiation. Based on previous studies of *P. gingivalis* infections, toll-like receptor 2/4 and nucleotide-binding oligomerization domain (NOD)-containing protein-like receptors were activated and mediated signaling pathways such as NF-κB and ERK1/2, which resulted in the overexpression of *IL6* (29–31). In addition to these previously identified mechanisms, our results revealed that *CEBPB* was activated as an upstream regulator of *IL6* (Figure 3B). CCAAT enhancer binding protein beta (*CEBPB*) is a transcription factor that plays an important role in immune and inflammation responses (32). *CEBPB* has been studied in the context of cellular transformation and cancer and was recently regarded as one of the master regulators in cancer biology, especially in mesenchymal glioblastoma (33). Importantly, a data mining study concluded that *IL6* and *CEBPB* were up-regulated in late stage OSCC. Similar to our results, Bai et al. (34) reported that up-regulation of *CEBPB* in human periodontal ligament cells promoted the production of *IL6* in response to the stimulation of lipopolysaccharide derived from *P. gingivalis*. However, direct regulation of *IL6* expression by *CEBPB* and its effect in OSCC needs to be validated.

As reported previously, a carcinogen induced oral carcinoma model revealed that *P. gingivalis* and *Fusobacterium nucleatum* promoted OSCC progression with the activation of *IL6*-*STAT3* (17). Interestingly, *IL6* was also predicted to activate *STAT1* in the present study (Figures 3A,B). Signal transducer and activator of transcription 1 (*STAT1*) was a hub gene with the highest median rank according to the expression validation with clinical samples. *STAT1* is a critical transcription factor involved in regulating cellular responses to interferons (IFNs), cytokines, growth factors and hormones (35). The oncogenic role of *STAT1* in certain types of cancer including HNSCC has been described (36–38). Phosphorylation of genes such as *STAT1* was predicted to be regulated by *LYN* (Figure 7). *LYN* proto-oncogene (*LYN*), a hub gene that belongs to the Src family of kinases, was associated with various cellular functions such as cell proliferation, metabolism and differentiation. The presence of *LYN* kinase in the tumor microenvironment was regarded as a key component for tumor progression (39). Furthermore, *LYN* was considered as a target in both epithelial cells and stromal cells of HNSCC (40). Recently, *LYN* was found to be an independent biomarker of HNSCC and was correlated with poor survival. Upon validation using seven clinical datasets, we also confirmed the overexpression of *LYN* in OSCC (Figure 4). In this study, we speculated that phosphorylation of *STAT1* might be one of the functions of *LYN* in OSCC in response to *P. gingivalis* infection.

An immunosuppressive microenvironment involving the activation of *PD1*/*PDL1* signaling has been increasingly considered to be important for tumor initiation and progression. As a critical mediator that was overexpressed in various types of cancer, *PD-L1* (programmed death-ligand 1, shown as *CD274*, also known as *B7H1*) was also observed to be up-regulated in OSCC cells by *P. gingivalis* infection, although the underlying mechanism was unclear (2, 41). In Figure 3A, *CD274* and its paralog *PDCD1LG2* (also known as *B7DC*) were regarded as targets of *STAT1*. Similarly, activation of *STAT1* was shown to mediate the high expression of *PD-L1* in HNSCC cells (36, 42). In addition, it was recently confirmed that IFNα, a cytokine belonging to the type I IFN family, induced overexpression of *PDL1* through the activation of *STAT1* (42). Interestingly, several type I IFN inducible genes such as *OAS1* (2'-5'-oligoadenylate synthetase 1) were also identified in the present study as hub genes and were regulated by *STAT1* (Figures 1, 3), which to some extent indicated the activation of endogenous IFNα (42). Taken together, we hypothesized that chronic infection by *P. gingivalis* contributed to an immunosuppressive microenvironment by targeting *CD274* and *PDCD1LG2* through the activation of *STAT1*.

*IL6* was also predicted to activate *BDNF* (Figure 3A). Brain derived neurotrophic factor (*BDNF*) was of great interest as it was predicted to be associated with tumorigenic properties (tumor cell proliferation, migration and invasion) as well as inflammatory response (Figures 3B,C). Though *BDNF* belongs to the nerve growth factor family, it is also expressed in tissues outside the nerve system. *BDNF* has been shown to be overexpressed in neoplastic specimens in various types of tumors including HNSCC (43, 44). Stimulation of *BDNF in vitro* promoted the invasive properties of HNSCC cells (43), which was similar to our results. As previously reported, within an inflammatory microenvironment, production of *BDNF* was enhanced in response to pro-inflammatory cytokines such as *IL6* (45). Correa et al. (46) were the first to report higher levels of *BDNF* in chronic periodontitis compared to control samples. However, it was recently suggested that recombinant human *BDNF* facilitated periodontal treatment by reducing excess inflammation (47). Thus, the functions of *IL6* and *BDNF* in OSCC upon *P. gingivalis* infection require further exploration.

In addition to *IL6, C3* was also a direct target of *CEBPB*, as predicted by IPA (Figure 3B). Complement system, the first defense against pathogens, is closely associated with tissue homeostasis. In the present study, Complement component 3 (*C3*) was considered as an upstream regulator and associated with inflammatory response. *C3* was also predicted to activate genes such as *IL6*. The undesirable activation of complement results in pathogenesis of inflammatory diseases as well as cancer (24). Interestingly, apart from immune cells such as macrophages, epithelial cells also secrete complement proteins. In neoplastic tissues, *C3* derived from tumor cells promoted tumor development by increasing cell proliferation (48). Several studies revealed the clinical role of *C3* as a salivary or sera biomarker of OSCC (49–51). However, the relationship between *P. gingivalis* and *C3* activation is rather complex and depends on the concentration of gingipains. *C3* is specifically activated when the concentration of gingipains is low (52). In the present cellular model, *C3* may be activated due to the low MOI of *P. gingivalis*, which is consistent with the low concentration of gingipains (52). Thus, we speculate that the constant activation of *C3* which might be mediated by *CEBPB* during *P. gingivalis* infection at a low MOI may promote the tumorigenic properties of HIOECs. Furthermore, potential interaction between *IL6* and *C3* within the inflammatory microenvironment remain to be investigated. Finally, other targets of *CEBPB*, which might be associated with OSCC development under *P. gingivalis* infection, should be explored in future studies.

As shown in Figure 8, some chemokines were identified as downstream molecules in the network. Chemokines have complex roles in tumor biology. Indeed, certain chemokines that were overexpressed in a paracrine method were associated with tumor suppression and improved tumor prognosis owing to the recruitment of favorable immune cell populations (53). However, similar to the present cellular model, chemokines such as *CXCL9, CXCL10, CXCL11* secreted in an autocrine manner by tumor cells were considered to contribute to a pro-tumoral microenvironment (25, 54). Thus, chemokines and their receptors are suggested to facilitate tumor initiation and progression and have been considered as key therapeutic targets for cancer (55, 56). *CXCL10* was one of the hub genes with a high fold change (shown in dark red). From an integrated microarray analysis, *CXCL10* was identified as a hub gene of periodontitis (57). As reported previously, *CXCL10* was associated with tumor cell motility and metastasis in various type of cancer (56, 58). Meanwhile, other chemokines including *CXCL2, CXCL11*, and *CXCL16* were also predicted to be activated downstream. A bioinformatics analysis suggested that *CXCL10* and *CXCL2* were members of the key molecules of epithelia in tongue squamous cell carcinoma (59). Overexpression of *CXCL11* was observed in the premalignant stage of OSCC, which was thought to be involved in carcinogenesis of OSCC (60). Though few studies have focused on the role of *CXCL16* in oral cancer, the promoting effect of *CXCL16* in tumor progression was observed in other types of cancer (61, 62). Future work on malignant tumor properties associated with chemokines in response to *P. gingivalis* infection may shed light on the transition of periodontitis to OSCC.

We acknowledge some limitations of our present work. In this study, DEGs in response to repeated infection by *P. gingivalis* for 15 weeks were shown and candidate genes associated with inflammation and tumorigenic properties were analyzed. However, additional studies should be applied to explore the exact roles of the identified molecules in OSCC and to validate the proposed interactions. In addition, though we validated the expression of hub genes in some clinical datasets of OSCC, other datasets derived from larger-scale clinical samples which contain information on periodontal conditions and prevalence rates of *P. gingivalis* should be applied for further validation and evaluation.

In conclusion, we identified *IL6, STAT1, LYN, BDNF, C3, CD274, PDCD1LG2*, and *CXCL10* as important candidates associated with OSCC in the present model and made attempts to illustrate the promoting role of *P. gingivalis* infection in OSCC initiation and progression. We suggested that chronic infection by *P. gingivalis* caused secretion of cytokines such as *IL6* to initiate OSCC and to activate tumorigenic transcription factors such as *STAT1* for tumor progression. During this complex process, overexpression of chemokines (such as *CXCL10*) may be involved in a positive loop of sustained tumor-associated inflammation. Immune escape of tumorigenic cells in response to *P. gingivalis* infection, regulated by *CD274*, should also be considered.

## Author Contributions

YP, FG, and QW contributed conception and design of the study. FG, CL, and JL analyzed the data. FG and QW wrote the draft of the manuscript. YP, DZ, and SZ revised the draft manuscript. All authors read and approved the submitted version.

### Conflict of Interest Statement

The authors declare that the research was conducted in the absence of any commercial or financial relationships that could be construed as a potential conflict of interest.
