# Supplementary material for: Identification of Potential Candidate Genes of Oral Cancer in Response to Chronic Infection With Porphyromonas gingivalis Using Bioinformatical Analyses
Source: Front Oncol. 2019 Feb 21;9:91. doi: 10.3389/fonc.2019.00091 (PMC6394248; doi:10.3389/fonc.2019.00091)
Supplement: Supplementary file 1 [file Image_1.pdf]

Supplementary Material

Identification of Potential Candidate Genes of Oral Cancer in Response to Chronic Infection with *Porphyromonas gingivalis* by Using Bioinformatical Analyses

Fengxue Geng[1] (co-first author), Qingxuan Wang[2] (co-first author), Chen Li[1], Junchao Liu[1], Dongmei Zhang[1], Shuwei Zhang[1], Yaping Pan[1*]

*** Correspondence:** Yaping Pan: yppan@cmu.edu.cn

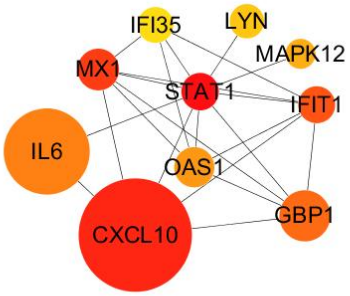

Rank	Node
1	STAT1
2	CXCL10
3	MX1
4	IFIT1
5	GBP1
6	IL6
7	OAS1
8	MAPK12
9	LYN
10	IFI35

Supplementary Figure 1

The hub genes were calculated and ranked with cytoHubba (Radiality).
